# BDCA2/FcɛRIγ Complex Signals through a Novel BCR-Like Pathway in Human Plasmacytoid Dendritic Cells

**DOI:** 10.1371/journal.pbio.0050248

**Published:** 2007-09-11

**Authors:** Wei Cao, Li Zhang, David B Rosen, Laura Bover, Gokuran Watanabe, Musheng Bao, Lewis L Lanier, Yong-Jun Liu

**Affiliations:** 1 Department of Immunology, University of Texas M. D. Anderson Cancer Center, Houston, Texas, United States of America; 2 Department of Biostatistics and Applied Mathematics, University of Texas M. D. Anderson Cancer Center, Houston, Texas, United States of America; 3 Department of Microbiology and Immunology, University of California at San Francisco, San Francisco, California, United States of America; 4 Biomedical Sciences Graduate Program, University of California at San Francisco, San Francisco, California, United States of America; 5 Cancer Research Institute, University of California at San Francisco, San Francisco, California, United States of America; Osaka University, Japan

## Abstract

Dendritic cells are equipped with lectin receptors to sense the extracellular environment and modulate cellular responses. Human plasmacytoid dendritic cells (pDCs) uniquely express blood dendritic cell antigen 2 (BDCA2) protein, a C-type lectin lacking an identifiable signaling motif. We demonstrate here that BDCA2 forms a complex with the transmembrane adapter FcɛRIγ. Through pathway analysis, we identified a comprehensive signaling machinery in human pDCs, similar to that which operates downstream of the B cell receptor (BCR), which is distinct from the system involved in T cell receptor (TCR) signaling. BDCA2 crosslinking resulted in the activation of the BCR-like cascade, which potently suppressed the ability of pDCs to produce type I interferon and other cytokines in response to Toll-like receptor ligands. Therefore, by associating with FcɛRIγ, BDCA2 activates a novel BCR-like signaling pathway to regulate the immune functions of pDCs.

## Author Summary

Dendritic cells (DCs) are specialized sentinels in the immune system that detect invading pathogens and, upon activation, initiate immune responses. DCs express C-type lectin receptors on their surface, which facilitate antigen capture. A distinct population of DCs, called plasmacytoid DCs (pDCs), display an extraordinary ability to rapidly make huge amounts of antiviral interferon (IFN) against viral infections. Human pDCs uniquely express a C-type lectin named BDCA2 that potently regulates pDCs function, yet the mechanism of how BDCA2 transduces signals is unknown. We show here that BDCA2 forms a complex with the transmembrane adapter FcɛRIγ. Using signaling pathway analysis, we discovered a comprehensive signaling machinery in human pDCs, similar to that which operates downstream of B cell receptors (BCRs), but distinct from the pathway involved in T cell receptor signaling. By associating with FcɛRIγ, BDCA2 activates a novel BCR-like signaling pathway to regulate the immune functions of pDCs. Since several pDC receptors use this pathway to modulate IFN and cytokine responses, these findings will guide more studies on how pDCs are regulated. Such mechanisms may lead to potential therapeutic interventions in autoimmune diseases involving hyperactivated pDCs, such as systemic lupus erythematosus and psoriasis.

## Abstract 3

Plasmacytoid dendritic cells (pDCs) are renowned for their production of type 1 interferon in response to viral infection, which is signified by Toll-like receptor (TLR) activation. Here, blood dendritic cell antigen 2(BDCA2), a C-type lectin receptor expressed uniquely on pDCs, is shown to block the ultimate effectors of TLR signaling via a novel pathway.

## Introduction

Dendritic cells (DCs) are specialized sentinels in the immune system that detect invading pathogens and pathological damages of the host. Upon activation, DCs instruct appropriate and effective immune responses [1–3]. Their extraordinary ability to capture antigens (self or foreign) is largely mediated by the collective expression of C-type lectin receptors (CLRs) on the cell surface [4,5]. In mouse spleen, DC subsets display a discrete expression profile of C-type lectins—CD8^+^ DCs express DEC-205 (CD205), whereas CD8^−^ DCs express dendritic cell immunoreceptor 2 (DCIR2), dendritic cell immunoactivating receptor (DCAR), Dectin-1, Dectin-2, DCIR3, and DC-SIGN [6]. This expression profile, in conjunction with the polarized expression of antigen processing and presentation machinery, contributes to the intrinsic functional difference between the DC subsets in regulating immunity [6]. In humans, C-type lectin ICAM3-grabbing nonintegrin (DC-SIGN, CD209) is expressed by mucosal DCs and langerin (CD207) is expressed by Langerhans cells [3,4]. Besides tissue DCs, macrophage, myeloid DCs (mDCs) and monocytes express a number of CLRs, such as Dectin-1, macrophage mannose receptor (MMR), and DCIR [3,4].

Plasmacytoid DCs (pDCs), also referred to as natural type I interferon (IFN)-producing cells (IPCs), are a distinct DC population with an extraordinary ability to rapidly produce massive amounts of type I IFN in response to viral infections [7,8]. Blood dendritic cell antigen 2 (BDCA2), a C-type lectin uniquely expressed on resting human pDCs, has been used to unequivocally identify pDCs in human peripheral blood and tissues [9]. It has an intracellular N terminus and only one extracellular carbohydrate recognition domain, which belongs to the type II C-type lectin group [5,10,11]. Similar to CLRs on other DC subsets, BDCA2 has been suggested to be capable of functioning as an antigen-uptake receptor, because monoclonal antibodies (mAbs) bound to BDCA2 on the surface of pDCs are efficiently internalized, and mAb-derived peptide bound to MHC class II can be presented to T cells [10].

Resting pDCs uniquely express high amounts of Toll-like receptor 9 (TLR9) and TLR7 [12–14], which recognize microbial DNA or RNA ligands in the endo-lysosomal compartment, respectively. After activation of TLR7 or TLR9, a multi-component “cytoplasmic transductional–transcriptional complex” is formed to initiate cellular activations [15–17]. Type I IFN production by pDCs has been shown to be independent of RNA helicases retinoic acid–inducible gene I *(RIG-I)*, melanoma differentiation-associated gene 5 *(MDA5)* and their adaptor IFN-ß promoter stimulator-1 *(IPS-1)* [15,18–20], which are essential for IFN response to viruses by other immune or nonhematopoietic cells. Therefore, pDCs carry out their unique innate immune functions through the TLR-mediated pathway. After exposure to viruses, pDCs differentiate into efficient antigen-presenting DCs and thus act as a critical mediator linking innate and adaptive immune responses against viral infections [7,8]. However, aberrant pDC activation in the absence of infection has been associated with autoimmune diseases. Patients with active systemic lupus erythematosus (SLE) have high amounts of type I IFN in their sera, which is likely due to the activation of pDCs by autoantibodies complexed with self nuclear antigens [21–24]. Because both TLR7 and TLR9 are located intracellularly, how pDCs sense the extracellular microenvironment and, in turn, modulate these TLR-mediated responses remains an important question.

BDCA2 was the first receptor described that negatively regulates the IFN response of pDCs, which is induced by CpG oligonucleotide, influenza virus, or by autoantibodies complexed with DNA [25,26]. However, it is not clear how BDCA2 modulates the TLR signaling, because its cytoplasmic domain does not contain any known signaling motifs, even though BDCA2 ligation by mAbs causes protein tyrosine phosphorylation and calcium influx [25]. A recent analysis of the closely related dendritic cell immunoreceptor family lectins, which include DCIR, DCAR, Dectin-2, and BDCA2, suggests a potential shared signaling mechanism as a result of the similar arrangement of their transmembrane domains [11,27,28]. Both DCAR and Dectin-2 have been shown to associate with FcɛRIγ to transduce cellular activation signals [29,30].

Here we report that BDCA2 signals in pDCs by associating with the transmembrane adapter FcɛRIγ and initiates an immunoreceptor-based tyrosine activation motif (ITAM)–dependent signaling cascade. We also provide evidence that human pDCs express a series of signaling molecules known to be specifically involved in BCR activation. We demonstrate that the BDCA2 and FcɛRIγ complex activates a prominent BCR-like signaling pathway in human pDCs to modulate the TLR-mediated responses.

## Results

### BDCA2 Associates with and Signals through FcɛRIγ

**Figure 1:**
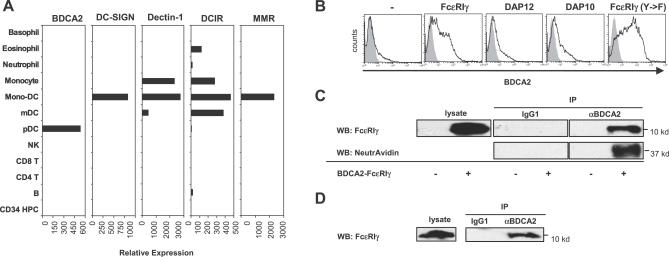
BDCA2 Associates with FcɛRIγ To Form a pDC-Specific Receptor Complex (A) The expression profile of BDCA2 transcripts in peripheral blood leukocytes by microarray gene expression analysis is shown. In comparison, the relative expression levels of DC-SIGN, Dectin-1, DCIR, and MMR are also shown. A value of <1 (arbitrary units) indicates the absence of gene expression. (B) Surface expression of BDCA2 after transduction of BDCA2 into Jurkat cells previously transfected with control, FcɛRIγ, DAP12, DAP10, or a Y-F ITAM mutant FcɛRIγ. The surface staining by isotype-matched control mAb is shown as a shaded area. (C) Co-immunoprecipitation of BDCA2 and FcɛRIγ from BDCA2 and FcɛRIγ doubly transfected Jurkat cells after cell surface biotinylation. Western blot analysis was performed with anti-FcɛRIγ Ab or with HRP-conjugated NeutrAvidin. (D) Co-immunoprecipitation of BDCA2 and FcɛRIγ from freshly isolated pDCs. Western blot analysis was performed with anti-FcɛRIγ Ab.

To determine the expression profile of BDCA2 in human leukocytes, we searched our established gene expression database, which includes the major immune cell types in peripheral blood. Strikingly, BDCA2 transcripts were expressed abundantly and exclusively by human pDCs (Figure 1A), consistent with its specific surface expression on pDCs within total peripheral blood mononuclear cell (PBMC) [9]. By contrast, immature peripheral blood CD11c^+^ mDC, monocyte, and monocyte-derived DC (mono-DC) expressed transcripts of other C-type lectins, such as DC-SIGN, Dectin-1, DCIR, and MMR, which were absent in pDCs (Figure 1A).

pDCs express two transmembrane adapter proteins bearing ITAMs, i.e., FcɛRIγ and DAP12, as well as DAP10, which signals via a YINM motif to activate PI3K [31]. To test whether BDCA2 interacts with any of these adapters, the individual adapters were first stably transduced into Jurkat cells, a human T cell line. When BDCA2 was transfected into Jurkat cells containing FcɛRIγ, the expression of BDCA2 on the cell surface was greatly enhanced, indicating a potential interaction between BDCA2 and FcɛRIγ (Figure 1B). In contrast, the presence of DAP12 and DAP10 only marginally affected BDCA2 expression, whereas both DAP12 and DAP10 increased the surface expression of mouse NKG2D, used as a positive control [31]. The enhanced BDCA2 surface expression in the presence of FcɛRIγ was also observed in several T and B cell lines (Figure S1). To demonstrate directly the physical association between BDCA2 and FcɛRIγ, we performed co-immunoprecipitation experiments and showed that FcɛRIγ existed in a complex that could be precipitated by antibody to BDCA2, but not by an isotype-matched control antibody from transfected Jurkat cells (Figure 1C) and, furthermore, from freshly isolated human pDCs (Figure 1D). These data suggest that BDCA2 and FcɛRIγ form a receptor complex with signaling potential.

**Figure 2:**
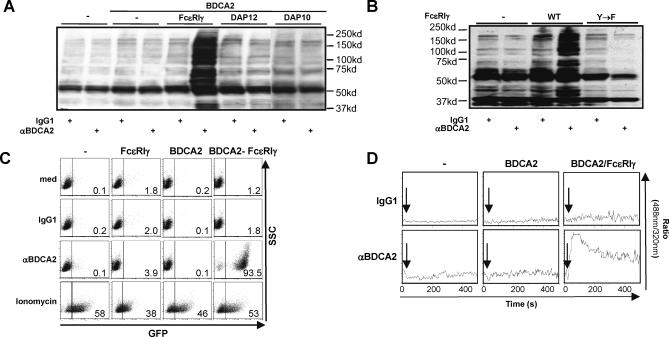
Crosslinking the BDCA2/FcɛRIγ Complex Transduces Activation Signals (A) Total protein phosphorylation in Jurkat cells transfected with BDCA2 in the absence or presence of adapter proteins by anti-BDCA2 mAb or control IgG1 crosslinking. Western blot analysis was performed with anti-phosphotyrosine mAb PY20. (B) Total protein phosphorylation induced by anti-BDCA2 mAb crosslinking in Jurkat cells transfected with BDCA2 and wild-type (WT) or mutant (Y-to-F) FcɛRIγ. (C) Transfected mouse 2B4 T hybridoma cells with an NFAT-GFP reporter (transgenes are labeled on the top) were crosslinked with control mouse IgG1 or anti-BDCA2 mAb. Cells were analyzed for GFP expression by flow cytometry. Cells cultured in medium alone or with ionomycin (positive control) were also analyzed. (D) The kinetics of intracellular calcium flux in transfected Jurkat cells (transgenes are labeled on top) when crosslinked (marked by arrow) by control (IgG1) or anti-BDCA2 mAb.

Previously, BDCA2-transfected Jurkat cells were shown to be unable to initiate protein tyrosine phosphorylation after BDCA2 crosslinking, an observation suggesting the potential involvement of a signaling adapter [25]. When BDCA2 was crosslinked on the Jurkat cells that were cotransfected with different adapters, Jurkat cells expressing FcɛRIγ, but not DAP12 or DAP10, prominently phosphorylated a spectrum of proteins (Figure 2A). We constructed a mutant FcɛRIγ in which the tyrosine (Y) residue within the intracellular ITAM was replaced with phenylalanine (F), rendering it unable to recruit downstream molecules. This mutant FcɛRIγ supported the elevated levels of surface expression of BDCA2 (Figure 1B) but was unable to mediate the protein tyrosine phosphorylation after BDCA2 crosslinking (Figure 2B), indicating an essential role of the ITAM in FcɛRIγ for the signaling by the BDCA2/FcɛRIγ complex.

Furthermore, when a human BDCA2/FcɛRIγ complex was introduced into a mouse T cell hybridoma line that contained an intracellular nuclear factor of activated T cells–green fluorescent protein (NFAT-GFP) reporter construct [31,32], crosslinking BDCA2 by mAb resulted in GFP expression (Figure 2C), indicating that the BDCA2/FcɛRIγ receptor complex was able to activate NFAT, similar to the effect of TCR-induced activation. In the absence of FcɛRIγ, crosslinking BDCA2 did not induce GFP expression (Figure 2C). Finally, calcium influx, one of the important cellular activation events that occurs downstream of ITAM signaling, was effectively triggered by BDCA2 crosslinking in Jurkat cells transfected with both BDCA2 and FcɛRIγ, but not in cells transfected with BDCA2 alone (Figure 2D), further confirming the requirement of FcɛRIγ for BDCA2 to signal.

### Human pDCs Express Signaling Molecules Involved in BCR Activation

To further delineate the intracellular signaling events following BDCA2 and FcɛRIγ activation, we examined the expression by pDCs of components known to be involved in ITAM-mediated signaling. In lymphocytes, several well-established signaling pathways exist downstream of the B cell and T cell antigen receptors, linked through ITAM-bearing subunits of these receptor complexes. We analyzed the expression of molecules involved in BCR and TCR activation in pDCs in comparison with peripheral B cells, T cells, natural killer (NK) cells, mDCs, and monocytes.

**Figure 3:**
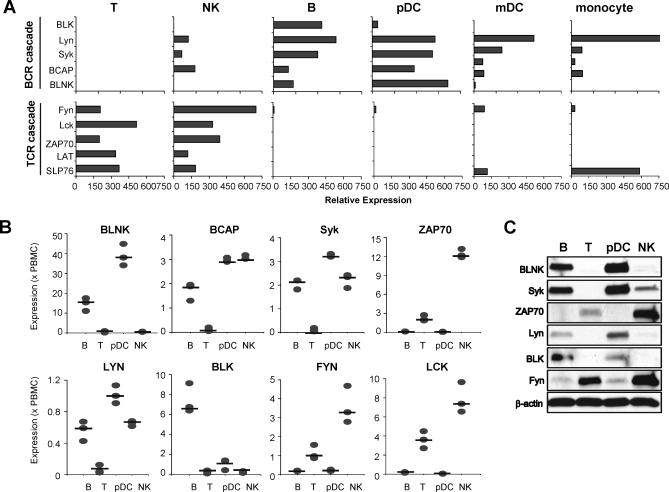
Human pDCs Express a BCR-Like Signaling Cascade (A) Expression of genes encoding molecules involved in BCR (top panel) and TCR (bottom panel) signaling in six peripheral leukocyte cell types from microarray gene expression analysis. (B) The relative gene expression of signaling molecules in different cell types from three healthy donors determined by quantitative RT-PCR analysis. The expression was normalized with the level of the transcript in total PBMC. The median expression is marked by a horizontal bar. (C) The expression of signaling proteins in different cell types was determined by Western blot analysis. The results from one representative donor out of three healthy donors analyzed are shown.

After receptor activation, two core tyrosine residues within the ITAM(s) are phosphorylated by Src family protein tyrosine kinases (PTKs). The phosphorylated tyrosines within the ITAMs associate with the src homology 2(SH2) domains of Syk-family PTKs, which in turn phosphorylate cell-type–specific intracellular adapters to initiate a multitude of signaling events. Each type of lymphocyte expresses and uses a distinct set of proteins to carry out the receptor-proximal signal transduction [33,34]. B cells express B lymphoid tyrosine kinase (BLK), Lyn kinase, spleen tyrosine kinase (Syk), and two adapter molecules, BLNK and BCAP, whereas T cells preferentially use two other Src-family PTKs, Fyn and Lck, and CD3ξ-associated protein kinase (ZAP-70) from the Syk-family of PTKs, as well as alternative adapters, i.e., SLP-76 and linker for activation of T cells (LAT) (Figure 3A). Strikingly, pDCs express many members of the BCR signaling cascade, but not those involved in TCR activation (Figure 3A). Of note, the levels of the B cell–specific adapter B cell linker (BLNK, also known as SLP-65 or BASH) was transcribed at higher levels in pDC than in B cells (514 relative units versus 133 relative units, respectively). Another B cell adapter, BCAP, which mediates phosphoinositide-3-kinase (PI3K) signaling [35], was also abundantly expressed by pDCs (Figure 3A). pDCs expressed lower levels of BLK compared to B cells. Interestingly, NK cells, in addition to expressing several members of the TCR signaling pathway, were found also to express several molecules involved in BCR signaling, e.g., Lyn, Syk and BCAP. mDC and monocyte similarly express Lyn, Syk, BCAP, and Fyn as well as the T cell adaptor SLP76. However unlike pDCs and B cells, they both lack the expression of BLNK and BLK (Figure 3A). Within all the peripheral cell leukocytes we examined, BLNK transcripts were only transcribed by pDCs and B cells (unpublished data).

We next analyzed the expression of the signaling components operating downstream of the molecules described above. pDCs and B cells selectively transcribed PLCγ2, Vav2, and Vav3, which are absent in T cells (Figure S2). On the other hand, high levels of PKCθ are abundantly expressed by T and NK cells, but not by pDCs or B cells (Figure S5). In contrast, the expression of other downstream signaling molecules, such as PI3K, Nck, MAP kinases, and NFAT, failed to show a distinct cell type–specific pattern (Figure S2).

**Figure 4:**
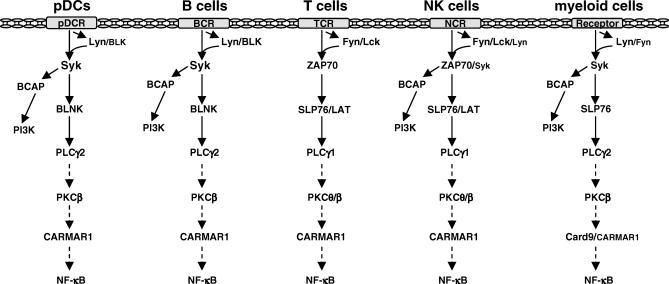
Diagram of ITAM-Mediated Signaling Pathways in Human Leukocytes The schematic cascades that are involved in receptor proximal signaling (solid arrows) and those leading to NF-κB activation (dotted lines) are shown.

We confirmed the transcription and protein expression of the BCR proximal signaling molecules in pDCs from several healthy donors by RT-PCR and Western blot analysis (Figure 3B and 3C). Taken together, our analysis revealed the existence of a potential ITAM-mediated signaling cascade in human pDCs similar to that of B cells, but distinct from that in T cells, NK cells, monocytes, or mDCs (Figure 4).

### BDCA2/FcɛRIγ Complex Transduces Signals via a BCR-Like Signaling Cascade

**Figure 5:**
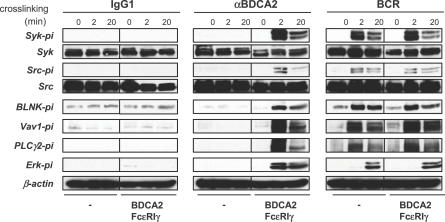
BDCA2/FcɛRIγ Complex Signals through the BCR Signaling Cascade in Transfected B Cells Shown are the kinetics of tyrosine phosphorylation of BCR intracellular signaling components in transduced Namalwa cells when crosslinked by control IgG1 or anti-BDCA2 mAb in comparison with BCR activation by anti-human Ig Ab.

To investigate whether the BDCA2/FcɛRIγ receptor complex could signal through the BCR signaling cascade, Namalwa cells, a human Burkitt lymphoma cell line, was transfected with BDCA2 and FcɛRIγ. Similar to BCR activation, crosslinking of the BDCA2/FcɛRIγ complex on Namalwa cells resulted in rapid phosphorylation of residue Y525 of Syk and residue Y416 of Src-family PTKs (Figure 5). After BCR activation, five tyrosine residues on BLNK are phosphorylated, enabling its function as a scaffold to integrate and propagate signals to downstream proteins [36]. BDCA2 ligation resulted in phosphorylation of residue Y84 of BLNK (Figure 4). Additional downstream molecules, such as Vav1 and PLCγ2, were phosphorylated by BDCA2 crosslinking, similar to BCR activation (Figure 5). MAP kinases Erk1/2, which are also activated after BCR engagement through the Ras-Raf pathway, were rapidly phosphorylated after BDCA2 stimulation (Figure 5). Furthermore, we observed a robust Ca^++^ influx in Namalwa cells after BDCA2 crosslinking, which is dependent on FcɛRIγ (unpublished data).

**Figure 6:**
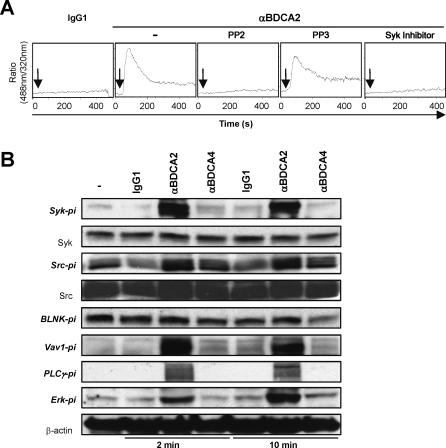
BDCA2/FcɛRIγ Complex Signals through a BCR-Like Signaling Cascade in Primary pDCs (A) The kinetics of intracellular calcium flux in freshly isolated pDCs when crosslinked (marked by arrow) by control IgG1 or anti-BDCA2 Ab. As indicated, cells were pre-incubated with PP2, PP3, or Syk inhibitor prior to crosslinking. (B) The kinetics of tyrosine phosphorylation on BCR intracellular signaling components in freshly isolated pDCs when crosslinked by control IgG1, anti-BDCA2 mAb, or anti-BDCA-4 mAb.

To study the signaling of the BDCA2/FcɛRIγ complex in primary human pDCs, we crosslinked BDCA2 by mAbs on freshly isolated pDCs and confirmed that it potently induces a transient intracellular calcium flux (Figure 6A). This activity was inhibited by PP2, a compound that inhibits Src family PTKs, but was not affected by an inactive control compound, PP3. In addition, Syk inhibitor completely abolished the intracellular calcium flux in BDCA2-crosslinked pDCs (Figure 6A). These results suggest the functional involvement of both Syk and Src-family PTKs during BDCA2 signaling. When the phosphorylation status of key signaling molecules in pDCs was analyzed, BDCA2 crosslinking resulted in rapid phosphorylation of both Src family PTKs and Syk (Figure 6B), indicating the onset of ITAM-induced signaling in pDCs. Different from the results with the Namalwa cell line, BDCA2 crosslinking did not enhance the pre-existing phosphorylation of BLNK in pDCs (Figure 6B, lane 5). High levels of phosphorylation of Y84 in the BLNK protein have been observed in freshly isolated, resting pDCs from several healthy donors, but not in freshly isolated human B cells (unpublished data). Moreover, downstream molecules, including Vav1, PLCγ2, and Erk1/2, were phosphorylated by BDCA2 crosslinking on pDCs (Figure 6B). In contrast, crosslinking with neither the isotype-matched control antibody nor the mAb against BDCA-4, another surface molecule expressed on pDCs, phosphorylated these proteins under the same conditions (Figure 6B). It should be noted that BDCA-4 beads were used to isolate pDCs, therefore BDCA-4 may no longer available for efficient crosslinking. The Src PTK inhibitor PP2 or Syk inhibitor, but not PP3, greatly diminished BDCA2-induced phosphorylation of both Src family PTKs and Syk and blocked the phosphorylation of Vav1 and PLCγ2 (Figure S3). Thus, the BDCA2/FcɛRIγ complex signals through a BCR-like signaling cascade in primary human pDCs.

### BDCA2/FcɛRIγ Complex Signaling Inhibits TLR-Mediated Type I IFN Production

**Figure 7:**
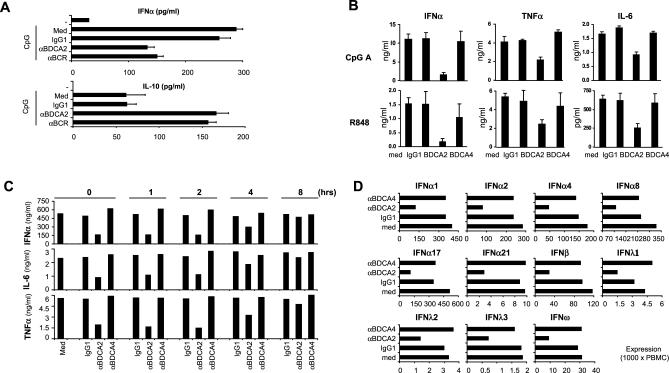
Ligation of the BDCA2/FcɛRIγ Complex Inhibits TLR-Induced Type I IFN Production (A) Transfected Namalwa cells were stimulated with CpG ODN and ligated with anti-BDCA2, anti-BCR, or control mAbs. Shown are the amounts of IFNα and IL-10 secreted by the cells stimulated CpG in a triplicate assay. (B) Purified pDCs were stimulated by CpG (top panel) or R848 (bottom panel) following anti-BDCA2 or control mAb crosslinking. The amounts of the secreted IFNα, TNFα, and IL-6 in a triplicate assay from a representative donor (*n* ≥ 3) are shown. (C) Purified pDCs stimulated by CpG first and then treated with anti-BDCA2 or control mAbs at different time intervals. The amounts of the secreted IFNα, TNFα, and IL-6 from a representative donor (*n* ≥ 3) are shown. (D) The amounts of type I IFN transcripts were determined by quantitative RT-PCR analysis. The expression is shown as the relative level of transcription compared with unstimulated PBMC.

Although BCR signaling synergizes with TLR triggering for optimal B lymphocyte activation [37], the impact of this pathway on TLR-mediated type I IFN responses needs to be defined. To accomplish this, we stably transfected human Burkitt lymphoma Namalwa cells with IRF7, BDCA2 and FcɛRIγ. These cells with endogenous TLR9 expression [38] produced IFNα in response to stimulation with CpG oligonucleotide (ODN) (Figure 7A). Crosslinking of BDCA2/FcɛRIγ or BCR together with CpG activation in these Namalwa cells resulted in enhanced interleukin 10 (IL-10) production, as expected, but decreased IFNα secretion (Figure 7A), indicating a selective suppressive effect by BCR or BDCA2 activation on TLR-induced IFNα production.

To investigate the function of the BCR-like signaling cascade in pDCs, we analyzed, in detail, the impact of BDCA2 crosslinking on the innate immune responses of human primary pDCs. Purified pDCs were stimulated with CpG ODN 2216 or R848, ligands of TLR9 and TLR7 or TLR8, respectively, in the presence of anti-BDCA2 mAb or control mAbs. BDCA2 ligation potently inhibited IFNα production and invariably reduced the production of tumor necrosis factor α (TNFα) and IL-6 induced by stimulation with these TLR ligands (Figure 7B). pDCs secreted lower levels of IFNα after BDCA2 ligation, but normal amounts of chemokines CXCL10/IP-10, CCL3/MIP-1α and CCL4/MIP-1β in response to CpG (Figure S4).

To evaluate the kinetics of BDCA2-mediated suppression of IFN production, pDCs were first stimulated with CpG and control or anti-BDCA2 mAbs were added to the culture at different times afterward. Strikingly, up to 4 h after pDCs were exposed to CpG ODN, crosslinking BDCA2 by mAb effectively inhibited IFNα and cytokine production by pDCs (Figure 7C). This result demonstrates the potency of BDCA2 to inhibit the TLR-induced innate immune responses by pDCs. Independently, pDCs from multiple donors were stimulated simultaneously with both CpG and anti-BDCA2 or control mAbs and at different lapsed times, the amounts of IFNα in the supernatant were measured (Figure S5). At 4 h after CpG treatment, pDCs treated with anti-BDCA2 mAbs reduced the production of IFNα by 96%. By 8 h post CpG treatment, BDCA2-ligated pDCs produced an average of 25.6% of IFNα compared to the amount made by control IgG1-incubated pDCs (Figure S5). Therefore, BDCA2 crosslinking likely blocked the initial induction of IFNα secretion.

To further determine the stage of IFN suppression, we measured the amount of type I IFN transcripts from CpG-activated pDCs that were crosslinked with anti-BDCA2 or control mAbs. BDCA2 crosslinking uniformly reduced the transcription of all the subtypes of type I IFN analyzed, i.e., IFNα, IFNβ, IFNγ, and IFNω (Figure 7D). Therefore, BDCA2 ligation blocks the transcriptional of type I IFN by pDCs in response to TLR activation.

## Discussion

To date, BDCA2 is the only C-type lectin that is found expressed exclusively on human pDCs, and it serves as a defining marker for pDCs. In addition, BDCA2 represents one of the most potent receptors that modulates the ability of pDCs to elicit type I IFN responses. Here we provide experimental evidence to reveal the signaling mechanism used by BDCA2 in human pDCs. On the cell surface, BDCA2 associates with FcɛRIγ to form a signaling receptor complex, which, upon ligation, activates a novel BCR-like intracellular signaling cascade, interfering with TLR activation.

ITAM-based signaling is the primary pathway of activation used by classical immunoreceptors, such as the antigen receptors on B and T lymphocytes, NK cell receptors, and the Fc receptors on myeloid cells [39–41]. ITAM-mediated signaling is also used by additional receptors, such as cell adhesion molecules, chemokine receptors, plexins, and lectin receptors [42]. pDC and mDCs represent two major subsets of professional antigen-presenting cells in human peripheral blood and tissues, which use different sets of pattern recognition receptors, such as TLRs, CLRs, and Nod-like receptors, for sensing and responding to microbial infections [43–50]. Our present study suggests that mDCs and pDCs not only express different repertories of lectin receptors, but also suggests that the signal pathways used by CLRs bearing ITAM elements on mDC and pDCs are intrinsically different. Ligation of either the BDCA2/FcɛRIγ complex or certain myeloid cell lectins, such as Dectin-1 and DC-SIGN [51,52], phosphorylates Syk, which is involved in BCR membrane-proximal signaling. However, downstream of Syk, pDCs use the intracellular adaptor molecule BLNK, whereas mDCs use SLP76 for amplification of signaling (Figure 7). In addition, recent studies showed that CARD9, a signaling adaptor molecule, plays a critical role in the signal transduction of Dectin-1 and ITAM-associated receptors in myeloid cells [53,54]. Our analysis suggests that similar to lymphocytes, pDCs do not express CARD9, but likely use CARMA1 (also named CARD11) to activate nuclear factor κB (NF-κB) [55–57] (Figure S6 and Figure 7).

Previous studies have shown remarkable similarities between B lineage cells and pDCs, including their plasma cell morphology and shared expression of several classes of genes. For example, they express a similar sets of TLRs (TLR7 and TLR9) [12,14,58], possess germ-line immunoglobulin (Ig) transcripts and Ig recombinase [59–61], have B cell–related transcription factor Spi-B [59,62], and use the same CIITA promoter for the controlling of MHC Class II expression [63]. The present demonstration that the pDC receptor BDCA2/FcɛRIγ and BCR share the same membrane-proximal signaling cascade and pathway leading to NF-κB activation provides further support for the intriguing relationship between pDCs and B lymphocytes.

A central mediator during B cell activation, BLNK, is phosphorylated by BCR activation on five tyrosine residues (Y72, Y84, Y96, Y178, and Y189) that serve as docking sites for recruiting downstream molecules [34,36]. Interestingly, in resting primary pDCs, we observed high levels of, if not saturating, phosphorylation of Y84 in BLNK, which is involved in PLCγ2 activation [36]. Further examination of tyrosine phosphorylation on other residues of BLNK may shed light on the dynamic involvement of this key molecule in nucleating signaling components during BDCA2 activation.

A recent study reported that BDCA2 may serve as an alternative receptor, in addition to CD4, CXCR4, and CCR5 on human pDCs, for HIV via binding to gp120 [64]. Therefore, it is plausible that HIV uptake by BDCA2 negatively affects the ability of pDCs to mount type I IFN responses. Similarly, Mycobacterium tuberculosis targets DC-specific C-type lectin DC-SIGN to infect DCs and to directly down-regulate DC-mediated immune responses [65]. Moreover, measles virus (MV) interacts with CD46, its cellular receptor on human monocytes, to specifically down-regulate IL-12 production, a mechanism causing MV-induced immunosuppression [66]. Therefore, BDCA2 may represent another example of antigen uptake receptors targeted by microbial ligands to negatively regulate cellular immunity.

Paradoxically, activation by antigens through the BCR triggers B cell proliferation, differentiation, and Ig and cytokine production, whereas crosslinking of BDCA2, which activates a similar BCR-like signaling pathway, fails to stimulate pDCs to proliferate or produce cytokines [10] (W. Cao, unpublished data), but rather inhibits the TLR-mediated responses. As multiple receptors on pDCs apparently use ITAM-based mechanisms to modulate type I IFN and cytokine responses during TLR activation [17,31,67–70], our findings will facilitate further investigations to determine how TLR activation is regulated in pDCs. Such mechanisms may provide opportunities for therapeutic interventions in autoimmune diseases, such as SLE [24], Sjögren syndrome [71,72], polymyositis [73,74], and psoriasis [75,76], where the causative mechanisms might involve hyper-activation of pDCs.

## Materials and Methods

### Media, cell lines, and reagents.

RPMI-1640 medium supplemented with 2 mM L-glutamine, 100 U/ml penicillin, 100 ng/ml streptomycin (Invitrogen; http://www.invitrogen.com), and heat-inactivated 10% FBS (Atlanta Biologicals; http://www.atlantabio.com) were used for the cell culture. The human acute T cell leukemia cell line Jurkat and the Burkitt lymphoma cell line Namalwa were purchased from the American Type Culture Collection (ATCC). The mouse 2B4 T hybridoma cell line was generously provided by H. Arase, Osaka University, Japan. CpG ODN 2216 (GGGGGACGATCGTCGG-GGGG and CpG 2006 (TCGTCGTTTTGTCGT-TTTGTCGTT) (Qiagen; http://www.qiagen.com) and R848 (InVivogen; http://www.invivogen.com) were used for pDC stimulation.

### Immune cell isolation and establishment of gene expression database.

The institutional review board for human research at the M.D. Anderson Cancer Center approved this study. Our gene expression database containing multiple types of immune cells isolated from human peripheral blood was established as described [31]. For this study, four peripheral leukocyte cell types were isolated simultaneously from buffy coats of individual healthy donors. Briefly, pDCs were positively selected from PBMC with anti-BDCA-4-coated microbeads and sorted by flow cytometry as CD3^−^CD4^+^CD8^−^CD11c^−^CD14^−^CD16^−^CD19^−^CD56^−^. The remaining PBMCs were stained and individual cell types were sorted accordingly: B cells (CD4^−^CD8^−^CD11c^−^CD14^−^CD16^−^CD19^+^CD56^−^CD235a^−^BDCA2^−^), CD4^+^ T cells (CD4^+^CD8^−^CD11c^−^CD14^−^CD16^−^CD19^−^CD56^−^CD235a^−^BDCA2^−^), and NK cells (CD3^−^CD4^−^CD8^−^CD14^−^CD19^−^CD56^+^CD235a^−^BDCA2^−^).

### Quantitative real-time PCR.

Total RNA extraction and quantitative real-time (RT)-PCR were performed as described [31]. Oligonucleotide primers used are listed in the Table S1 or described previously [31].

### Cloning and expression of BDCA2 and transmembrane adapters.

Full-length human BDCA2 was cloned from cDNAs generated from human peripheral blood pDCs by high-fidelity PCR using the following primers: sense, ATATGGATCCATGGTGCCTGAAGAA-GAGC and antisense, CTATGAATTCTTATATGT-AGATCTTCTTCATC. The BDCA2 cDNA was then cloned into the Bam HI and Eco RI sites of a lentiviral vector FG-30 [77]. Full-length cDNA encoding human FcɛRIγ, DAP12, and DAP10, as described previously [31], were subcloned into FG-30. An FcɛRIγ mutant with Y-to-F substitution in the ITAM was prepared by PCR and cloned into the FG-30 lentiviral vector for gene transduction.

### NFAT-GFP reporter cell assays.

Both BDCA2 and FcɛRIγ, or each cDNA individually, were transduced into mouse 2B4 T hybridoma cells stably expressing an NFAT-GFP reporter construct [32]. Transfected cells were cultured at 10^6^ cells/ml in the presence of 10 μg/ml of plate-bound anti-BDCA2 mAb AC144 (Miltenyi Biotec; http://www.miltenyibiotec.com) or purified IgG1 isotype-matched control mAb (eBioscience; http://www.ebioscience.com) for 20 h and then analyzed for GFP expression by flow cytometry.

### Cell surface biotinylation and protein immunoprecipitation.

2 × 10^7^ cells that were pre-washed with phosphate-buffered saline (PBS) were incubated with 2 mM EZ-Link Sulfo-NHS-LC-Biotin (Pierce Biotechnology; http://www.piercenet.com) according to manufacturer's protocol. Cells were then washed 3 time with 100 mM glycine in 1× PBS, and lysed in Brij-NP-40 lysis buffer (0.875% Brij 97, 0.125% Nonidet P-40, 10 mM Tris base, pH 8.0, 150 mM NaCl plus protease inhibitors). Then the lysates were cleared by a spin at 10,000*g* for 10 min. The lysates were incubated with either control mouse IgG1 or anti-BDCA2 mAb conjugated to Protein G-Sepharose (Pierce Biotechnology) at 4 °C overnight. The sepharose beads were washed extensively with the lysis buffer before elution of the antigen with SDS sample buffer. Western blots were performed with rabbit anti- FcɛRIγ antibody (gift from Dr. M-H Jouvin, BIDMC, Boston) and HRP-conjugated goat anti-rabbit IgG or HRP-labeled neutravidin (Pierce Biotechnology).

### Western blot analysis of protein tyrosine phosphorylation and expression.

5 × 10^5^ cells transfected with BDCA2 and FcɛRIγ were incubated with control mouse IgG1 (eBioscience) or anti-BDCA2 mAb (Miltenyi Biotec), and then crosslinked with F(ab')_2_ goat anti-mouse IgG (Jackson ImmunoResearch Lab; http://www.jacksonimmuno.com). BCR crosslinking was performed by adding 15 μg/ml F(ab')_2_ goat anti-human Ig (Southern Biotech). 5 × 10^5^ pDCs isolated using a BDCA-4 cell isolation kit (Miltenyi Biotec) were incubated with control mouse IgG1, anti-BDCA2, or anti-BDCA-4 mAb (Miltenyi Biotec). Western blot was performed with anti-phospho Src family Ab, anti-phospho Syk Ab, anti-phospho Erk1/2, anti-non-phospho Src family Ab, anti-Syk Ab (Cell Signaling Technology; http://www.cellsignal.com), anti-phospho BLNK, anti-phospho Vav1 (Abcam; http://www.abcam.com), anti-phospho PLCγ2 (BD Biosciences; http://www.bdbiosciences.com), and anti-β-actin mAb (Sigma; http://www.sigmaaldrich.com).

### Calcium influx assay.

10^6^ transfected cells or pDCs, pre-loaded with Fluo4- and Fura Red-AM (Invitrogen), were crosslinked as described above and analyzed on a FACSAria (BD Biosciences) and data were evaluated by using FlowJo software (TreeStar; http://www.treestar.com). As indicated, cells were pre-incubated for 30 min with 25 μM PP2, 25 μM PP3, or 5 μM Syk inhibitor (EMD Biosciences; http://www.emdbiosciences.com) prior to crosslinking.

### Namalwa transfectants.

Namalwa cells were transduced sequentially by lentiviral vectors expressing human IRF7, BDCA2, and FcɛRIγ. Full-length human IRF7 was cloned from cDNAs generated from purified human peripheral pDCs using a high-fidelity Taq polymerase (Invitrogen) with primers; sense, ACCTCTAGAATGGCCTTGGCTCCTGAGAGG, and antisense, ATTCTCGAGCTAGGCGGGCTGCTCCAGCTCC. The transduced Namalwa cells were stimulated with CpG ODN 2006 and then cultured in the presence of 10 μg/ml of plate-bound anti-BDCA2 mAb AC144, F(ab')_2_ goat anti-human Ig, or purified control IgG1 for 20 h. Supernatants were analyzed for cytokine secretion by ELISA using a human IFNα kit (Bender MedSystems; http://www.bendermedsystems.com) and IL-10 kit (R&D Systems; http://www.rndsystems.com).

### BDCA2 crosslinking on pDCs in culture.

5 × 10^4^ pDCs isolated by enrichment with anti-BDCA-4–coated microbeads and subsequent flow cytometry (CD4^+^CD11c^−^CD3^−^CD14^−^CD16^−^CD19^−^CD56^−^) were incubated with 5 μg/ml anti-BDCA2 or control mAb in 100 μl culture medium for 30 min prior to stimulation with 1 μM CpG 2216 or 0.05 μg/ml of R848. Cells and supernatants were harvested 18 h later for real-time quantitative PCR analysis and enzyme-linked immunosorbent assay (ELISA) using a human IFNα kit (Bender MedSystems), IFNα kit (PBL Biomedical Laboratories; http://www.interferonsource.com), IL-6 kit, TNFα kit, CXCL10 kit, CCL3 kit, and CCL4 kit (R&D Systems). For kinetic studies, pDCs were stimulated with 1 μM CpG 2216 and co-cultured with anti-BDCA2 or control mAbs at time 0. At different time points, supernatants were harvested for quantitation of cytokines or IFN by ELISA. Alternatively, CpG was added first to pDC cultures, and then anti-BDCA2 or control mAbs were added at different times thereafter. Eighteen h after addition of CpG, supernatants were harvested for quantitation of cytokines or IFN by ELISA.

## Supporting Information

Figure S1 Expression Levels of BDCA2 in the Presence and Absence of FcɛRIγ on Transfected T Cells (Mouse 2B4 T Hybridoma and Human Jurkat Cell Lines) and Transfected B Cells (Human HPB-Null Cell Line) (79 KB PPT)

Figure S2 Relative Transcription of Intracellular Molecules Involved in Antigen Receptor Signaling in Four Lymphoid Cell Types Based on Microarray Gene Expression Analysis (57 KB PPT)

Figure S3 Blocking of Tyrosine Phosphorylation of BCR-Like Intracellular Signaling Components Induced by BDCA2 Crosslinking by Inhibitors of Src PTK or Syk in Freshly Isolated pDCs (276 KB PDF)

Figure S4 Crosslinking of BDCA2 Inhibits TLR-Induced IFNβ but Not Chemokine Production Purified pDCs were stimulated by CpG following anti-BDCA2 or control Ab crosslinking. The amounts of secreted IFNβ, CXCL10/IP-10, CCL3/MIP-1α and CCL4/MIP-1β from a representative donor (*n* ≥ 3) are shown.
(49 KB PPT)

Figure S5 Purified pDCs Were Co-Stimulated by CpG and mAbs (Anti-BDCA2 or Control IgG) for 4 or 8 Hours The relative amounts of the secreted IFNα from four donors are shown. The median expression is marked by a horizontal bar. The numbers on top of the symbols indicate the average amounts of IFNα (pg/ml) present in the culture supernatant.
(44 KB PPT)

Figure S6 Relative Transcription of Intracellular Molecules Involved in NF-κB Activation in Peripheral Immune Cells Based on Microarray Gene Expression Analysis (49 KB PPT)

Table S1 Oligonucleotide Primers Used (26 KB DOC)

## Abbreviations

BCR: B cell receptor
BDCA2: blood dendritic cell antigen 2
CLR: C-type lectin receptor
DC: dendritic cell
DCIR: dendritic cell immunoreceptor
GFP: green fluorescent protein
IFN: interferon
ITAM: immunoreceptor-based tyrosine activation motif
mAb: monoclonal antibody
mDC: myeloid DC
NFAT: nuclear factor of activated T cells
NF-κB: nuclear factor κB
NK cell: natural killer cell
pDC: plasmacytoid DC
PTK: protein tyrosine kinase
TCR: T cell receptor
TLR: Toll-like receptor
