# Supplementary material for: BDCA2/FcɛRIγ Complex Signals through a Novel BCR-Like Pathway in Human Plasmacytoid Dendritic Cells
Source: PLoS Biol. 2007 Sep 11;5(10):e248. doi: 10.1371/journal.pbio.0050248 (PMC1971124; doi:10.1371/journal.pbio.0050248)
Supplement: Supplementary file 1 [file pbio.0050248.sg003.pdf]

supFig3

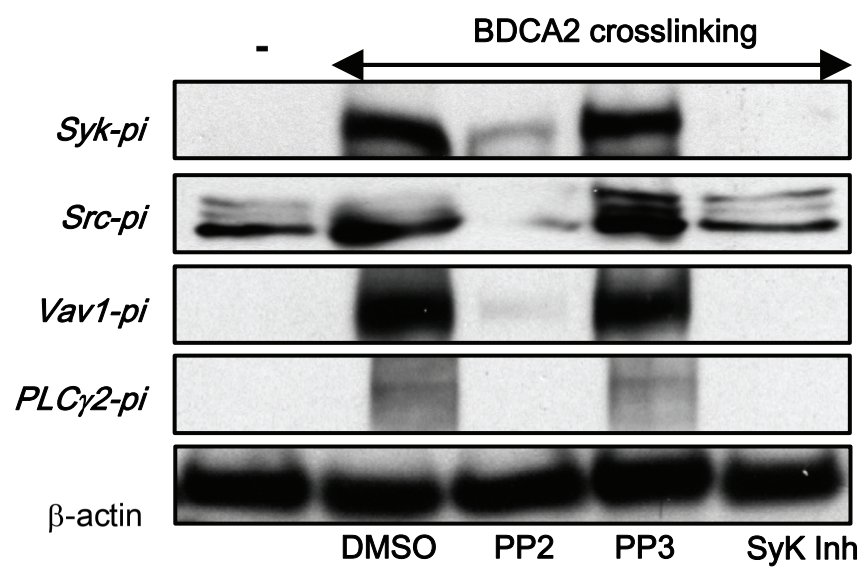

-
BDCA2 crosslinking
Syk-pi
Src-pi
Vav1-pi
PLCγ2-pi
β-actin
DMSO
PP2
PP3
SyK Inh
