# Supplementary material for: BDCA2/FcɛRIγ Complex Signals through a Novel BCR-Like Pathway in Human Plasmacytoid Dendritic Cells
Source: PLoS Biol. 2007 Sep 11;5(10):e248. doi: 10.1371/journal.pbio.0050248 (PMC1971124; doi:10.1371/journal.pbio.0050248)
Supplement: Supplementary file 2 [file pbio.0050248.st001.doc]

Gene	Forward primers	Reverse primers	
BLNK	CGGGCCGGGAAAAAAC	GCATTCTGTTGAGAGGCAACTG	
BCAP	TGAGGACGAGAAGGTTGTTTCC	GCCCCACAGCGAATGC	
Syk	CTGAAAAACGAGGCCAATGAC	GCTGCATGACATTTGCTTCTG	
ZAP70	GGTGCCGACGACAGCTACTAC	TTGCGGAAGTTGATGCATTC	
Lyn	CATCGAGCGGAAGAACTACATTC	GACTCGGAGACCAGAACATTAGC	
BLK	AGCAGACGTGTGGTCGTTTG	TGGCACCCGCCCATAAG	
FYN	CATCGAGCGCATGAATTATATCC	CAATCTTGCATATGAGTCCATTCC	
LCK	CGGGCTGCCAACATTCTG	GGCGTGCTAGGCCAAAGTC	
BTK	TCCAGGTATGTCCTGGATGATG	ACCACCGGACTGGAAATTTG	
IFNα8	TGATCCAGCAGACCTTCAACCT	GGTCTCATCCAAAGCAGCAGAT	
IFNα17	CCAGTTCCAGAAGACTCAAGCC	TGCTGGATCATCTCATGGAGG	
IFNα21	TCTGCCTCAGACCCACAGC	GCCAGGAGTATCAAGGCCC	
IFNλ1	TGTCACCTTCAACCTCTTCCG	GCCACATATTTGAGGTCTCGC	
IFNλ2	CCCCAAAAAAGGAGTCCCC	GGTTGAAGGTGACAGAGGCC	
IFNλ3	GGGATGTCTTGGACCAGCC	GCTGGGAGAGGATATGGTGC	
IFNω	AGACTTCAGGTTCCCCCAGG	GCCTTCTGCAACTGGCTCC
